# Real-world evidence from 50,000 online participants using MoCA-XpressO for cognitive prescreening

**DOI:** 10.1038/s41598-026-35640-0

**Published:** 2026-01-13

**Authors:** Willem Huijbers, Hans-Aloys Wischmann, Johanna Gruber, Kacylia Pistoia, Joana Krieger, Murray Gillies, Ziad Nasreddine

**Affiliations:** 1 MoCA Cognition, Montreal, QC Canada; 2 Institute of Public Health, Charité – Universitätsmedizin Berlin, Berlin, Germany

**Keywords:** Cognitive prescreening, Digital cognitive test, Alzheimer’s disease, Diseases, Health care, Medical research, Neuroscience, Psychology, Psychology

## Abstract

XpressO is a digital cognitive prescreening tool developed by Montreal Cognition (MoCA Test Inc.). In this study, we evaluated real-world online data collected through XpressO in 2024 and early 2025, based on over 50,000 self-enrolled online participants. In line with expectations, we found that the XpressO score—which predicts screening positive for (mild) cognitive impairment—is associated with sex, age, and education. The results indicated that women have a 5.8% [5.3%, 6.3%] lower relative risk of prescreening positive for cognitive impairment, each additional year of age increases the relative risk by 0.59% [0.57%, 0.60%], whereas each year of education decreases the relative risk by 0.99% [0.94%, 1.06%]. We also visualized and quantified interaction effects among these demographic variables as predictors of the XpressO score. While the interaction effect between sex and age was not statistically significant, all other interaction terms, including the three-way interaction between sex, age, and education, were significantly associated with the XpressO score. Additionally, adjusting for demographic factors reduced the observed effect of potential confounders, the language and the platform used. However, when we evaluated a score adjusted for demographics in a clinical cohort of 101 participants, we found that this adjustment slightly but significantly reduced the discriminatory power of the XpressO tool in identifying individuals with cognitive impairment from 0.86 to 0.81. These findings from a real-world online cohort offer novel insights into the complex influence of demographic factors on digital cognitive prescreening. Moreover, they demonstrate that XpressO is a viable tool for online prescreening and can help streamline the diagnostic pathway for individuals who may be eligible for disease-modifying treatments for Alzheimer’s disease.

**Supplementary Information:**

The online version contains supplementary material available at 10.1038/s41598-026-35640-0.

## Introduction

Mild Cognitive Impairment (MCI) remains severely underdiagnosed^1–3^. At the same time, many older adults are concerned about brain health, especially when they notice memory problems or subjective cognitive decline. This growing awareness coincides with an increasing prevalence of MCI and dementia in aging populations, which is placing considerable strain on primary care physicians, neurologists, and neuropsychologists^4,5^. As a result, healthcare providers are increasingly challenged, not only by the growing number of patients with cognitive disorders, but also by a surge of cognitively normal adults who are concerned and seek medical evaluations—the so-called “silver tsunami”^6^. Online cognitive prescreening holds the promise to prioritize individuals who may require further evaluation. This can facilitate earlier detection of neurological disorders, help streamline the diagnostics pathway, and reduce costs^7^.

The recent approval of anti-amyloid therapies for patients with MCI and mild dementia has increased the urgency to provide faster and more equitable access to diagnostic care, as these therapies are likely more effective when initiated in early stages of the disease^8,9^. Hence, there is a growing interest in unsupervised digital cognitive tests, which present unique opportunities for remote self-assessments to assist in diagnostic care^10–17^. However, most digital cognitive (pre)screening tests are only evaluated in a clinical cohort of relatively modest size, and only a few have been evaluated in larger online cohorts (e.g., Wu et al.^17^; Butler et al.^18^). Therefore, it remains unclear how demographic factors influence online cognitive prescreening results and there is no consensus whether a demographic correction affects the discriminative ability and algorithmic fairness in sensitivity and specificity across sex, age, and education strata^19,20^.

We therefore aimed to evaluate the influence of demographic factors on an online self-administered prescreening tool for MCI and mild dementia, called XpressO^21^. XpressO was developed by MoCA Test Inc. as a prescreener for the Montreal Cognitive Assessment (MoCA) test^22^. The tool generates a probabilistic score that predicts whether participants would screen above or below the cut-off for MCI on the MoCA^23^. By inference, a “high” XpressO score thus predicts that a participant is cognitively normal while a “low” XpressO score predicts MCI or mild dementia. XpressO was made available in early 2024. In little more than a year, over 50,000 unique participants completed at least one session. This self-enrolled cohort provided an opportunity for analysis of prescreening scores in a real-world setting. We aimed to assess associations between the XpressO score, sex, age, and education, and effects of two potential confounders, the language and platform used.

Based on the relation between XpressO and the MoCA test^21^, we hypothesized that low XpressO scores, and therefore increased prescreening rates for MCI, would be more prevalent among online participants with higher age^24–26^. Similarly, given the extensive literature on the protective effects of education^27,28^, we expected higher XpressO scores, and thus lower prescreening rates, among participants with more years of education. The literature on sex is slightly more ambiguous, as women have a greater overall, unadjusted risk for dementia, mostly related to longevity^29^. While most studies found higher rates of MCI for men when adjusted for age^30^, some studies found the opposite^31^. Nevertheless, women typically tend to outperform men on the MoCA test^26,32^. Thus, given the relation between XpressO and the MoCA test, we hypothesized that women would show higher XpressO scores. We also anticipated interactions between sex, age, and education based on studies that examined MoCA scores in age-matched groups^26,32^. Similarly, studies on the risk of Alzheimer’s disease identified interactions between sex and education^33^. Given the complexity of interactions and limited studies examining sex, age, and education together, we did not predict their direction nor magnitude.

Secondly, we hypothesized that a correction for demographic factors would decrease the diagnostic performance of XpressO. There is an ongoing debate as to whether cognitive assessment scores should be adjusted for demographic factors, including sex, age, and education, as multiple studies established adjustment formula or correction tables for the MoCA test scores^34–36^. It is often assumed that a demographic correction improves the discriminative ability^37^, when scores are standardized relative to a population of healthy peers. However, a recent simulation study confirmed that a correction is detrimental for the discriminative ability of screening tests, while improving consistency across demographic strata^20^. This is aligned with methodological studies that argued that a demographic correction is not always straight-forward, especially when demographic variables are not independent^38–40^. We therefore planned to use the online cohort to generate a logistic score adjusted for demographic covariates and evaluate the performance in a clinical cohort of 209 participants for whom a paper MoCA was collected as the ground truth (See Supplementary Table 1). Together, we conjectured that these analyses would shed light on the influence of sex, age, and education and on the potential benefit of a demographic adjustment.

## Methods

### Study design

We gathered real-world evidence in a large self-enrolled online cohort and controlled data in two clinical studies. The clinical studies were used to update and validate the XpressO score (see Supplementary Materials). In the second clinical study, used to evaluate the demographic adjustment, we included 101 patients and caregivers at the MoCA Clinic, and older adults from a retirement home near Montreal (see Supplementary Table 1). The studies adhered to the declaration of Helsinki. Informed consent was obtained from all participants of the clinical studies, and these studies received approval by the Optimum Ethics Review Board. The data from the online cohort was collected between January 2024 and March 2025. In that period 56,064 participants self-enrolled and provided consent for use of their anonymized data for research purposes [https://portal.mocacognition.com/privacy].

The real-world data from the XpressO prescreening task was gathered remotely and unsupervised via an application installed on a mobile device or within a web browser. In the clinical studies, participants first completed a paper MoCA test together with the rater, followed by the XpressO task in an unsupervised setting. The details of XpressO tasks have been described previously^21^, but in short: XpressO includes a placement task, a logical sequence task, and a delayed recall task. These sub-tasks are performed 3 times with various stimuli, and the entire test is typically finished within 5–7 min. After the test is completed, the tool generates a logistic score that predicts whether participants would score above or below the threshold for MCI on a MoCA test^23^. A low XpressO score is defined as a value of 42 or lower, high XpressO score corresponds to 72 or higher, and 43–71 reflects an intermediate range. The XpressO test was made available in early 2024 in the Apple App. Store [https://apps.apple.com/us/app/xpresso-by-moca/id6449499046] and Google App. Store [https://play.google.com/store/apps/details?id=com.moca.xpresso&pcampaignid=web_sharest] and is currently available in English, French, Spanish, Arabic, Italian, German, Japanese, and Turkish. In view of the more recent release of languages other than English and French, we only included results from online participants who conducted the test in French or English (96.98%). Also, to evaluate the influence of demographic factors, we excluded data from participants who did not disclose sex, age, or education (0.86%), entered an age under 18 or over 120 years (0.75%), or reported sex as “other” (1.27%). Finally, we capped years of education at a maximum of 30 years to reduce the influence of extreme values and excluded participants who reported too many years of education relative to age. Specifically, participants were excluded if their age was less than their years of education plus six (e.g. kinder garden). This quality criterion led to the exclusion of 1.57% of the online cohort.

This enabled us to analyze 52,147 participants from the online cohort. Demographic characteristics were described using the mean, standard deviation (SD), median, minimum (min), and maximum (max) for continuous variables, and percentages for categorical variables (Table 1). Education was quantified by years of education, and as a categorical variable consistent with the ISCED guidelines^41^. For visualization purposes, we combined the ISCED levels 0 and 1 into “years of education < 7 “, kept level 2 as “7–9 years “, level 3 as “10–13 years “, and level 4 as “14–15 years “, and merged levels 5–8 into “years of education ≥ 16”. For each participant, we only analyzed results from their first completed XpressO test (see Table 2).

**Table 1:** demographics of the online participants stratified by the categories of the XpressO score. Tables include number of analyzed participants (N), minimum (Min), maximum (Max) and standard deviation (SD).

	Low XpressO Score (N = 9,844)	Intermediate XpressO Score (N = 10,874)	High XpressO Score (N = 31,429)	Overall (N = 52,147)
Age (years)				
Mean (SD)	66.9 (12.5)	62.5 (13.0)	56.6 (13.9)	59.8 (14.1)
Median [Min, Max]	69.0 [18.0, 108]	65.0 [18.0, 93.0]	59.0 [18.0, 119]	62.0 [18.0, 119]
Sex				
Male	4762 (48.4%)	4478 (41.2%)	11,486 (36.5%)	20,726 (39.7%)
Female	5082 (51.6%)	6396 (58.8%)	19,943 (63.5%)	31,421 (60.3%)
Education (years)				
Mean (SD)	13.6 (4.47)	14.6 (4.16)	15.5 (3.87)	15.0 (4.12)
Median [Min, Max]	13.0 [0, 30.0]	15.0 [0, 30.0]	16.0 [0, 30.0]	15.0 [0, 30.0]
Language				
French	7088 (72.0%)	6817 (62.7%)	18,106 (57.6%)	32,011 (61.4%)
English	2756 (28.0%)	4057 (37.3%)	13,323 (42.4%)	20,136 (38.6%)
Platform				
Application	2246 (22.8%)	3335 (30.7%)	10,801 (34.4%)	16,382 (31.4%)
Web Browser	7598 (77.2%)	7539 (69.3%)	20,628 (65.6%)	35,765 (68.6%)
Recruitment Wave (year)				
2024	2497 (25.4%)	3761 (34.6%)	12,414 (39.5%)	18,672 (35.8%
2025	7347 (74.6%)	7113 (65.4%)	19,015 (60.5%)	33,475 (64.2%)
Memory tasks: Average number of same objects				
Mean (SD)	4.27 (0.715)	4.82 (0.209)	4.97 (0.0985)	4.81 (0.426)
Median [Min, Max]	4.33 [0, 5.00]	5.00 [2.67, 5.00]	5.00 [3.00, 5.00]	5.00 [0, 5.00]
Logical tasks: Average number of 100% correct answers				
Mean (SD)	3.74 (1.83)	5.20 (1.30)	6.98 (0.810)	6.00 (1.75)
Median [Min, Max]	3.67 [0, 7.67]	5.00 [1.67, 7.67]	7.00 [3.33, 7.67]	6.33 [0, 7.67]
Total time (minutes)				
Mean (SD)	5.97 (2.32)	5.46 (1.77)	4.64 (1.43)	5.06 (1.79)
Median [Min, Max]	5.52 [0.567, 19.6]	5.15 [1.25, 15.5]	4.43 [0.700, 15.0]	4.73 [0.567, 19.6]
XpressO Score				
Mean (SD)	19.2 (13.6)	58.8 (8.74)	89.9 (7.85)	70.1 (29.0)
Median [Min, Max]	19.1 [0, 42.0]	59.5 [42.0, 72.0]	92.0 [72.0, 100]	80.4 [0, 100]

### Statistical analyses

Statistical analyses and visualization were performed using R v4.4.2^42^, R-studio v2024.12.1 + 563 and the R-packages ggplot2 v3.5.1^43^, pROC v1.18.5^44^, Table1 v1.4.3^45^, stats v.4.4.2, ordinal v4.1, sandwich v3.1, lmtest v0.9 and marginaleffects v0.27.0^46^. We used linear models (LM) from the base R stats package to estimate the association with demographic factors and potential confounders. First, we estimated the un-adjusted (raw) effects and subsequently the adjusted effects using a linear model that simultaneously included sex (male or female), age (in years), and education (in years). Male was used as the reference group for sex. While the model included an intercept, we only reported the estimates (*β*) for the predictors, their 95-percent confidence intervals (CI_95_), Akaike Infromation Criterion (AIC) and the respective p-values and false-discovery rate (FDR) orrected p-values_FDR_. The CI_95_ were estimated using the Wald-type intervals (see also supplemental materials) We also estimated the marginal effects of sex, age, and education on the XpressO score, using a LM with all interactions terms between sex, age and education. We estimated the contrast across the levels of each variable, conditional on the others. We conducted two additional analyses to estimate the influence of language and the influence of platform. The mobile application was used as the reference group for platform, and French for language. We estimated the influence of these potential confounders before and after correcting the XpressO score for sex, age, and education. To clarify whether these performance differences were (partially) due to different demographics of the participants in the language or platform strata, we used a linear model that simultaneously estimated the effects of platform and language while controlling for the effects of age, sex, education and the interaction terms.

We also evaluated the influence of a demographic adjustment on prescreening performance. We used the online real-world cohort (*N* = 52,147) to determine normative adjustments and evaluated the performance of the corrected XpressO scores using the clinical validation cohort (*N* = 101). See Supplementary Materials for demographic characteristics of the clinical cohorts. We estimated the area under the curve (AUC) based on the un-adjusted XpressO score, based on an XpressO score adjusted only for the main effects of sex, age, and education, and finally based on an XpressO score adjusted for both the main effects and all interaction terms. We compared the AUCs from the unadjusted and adjusted scores using a DeLong test.

## Results

**Fig. 1:**
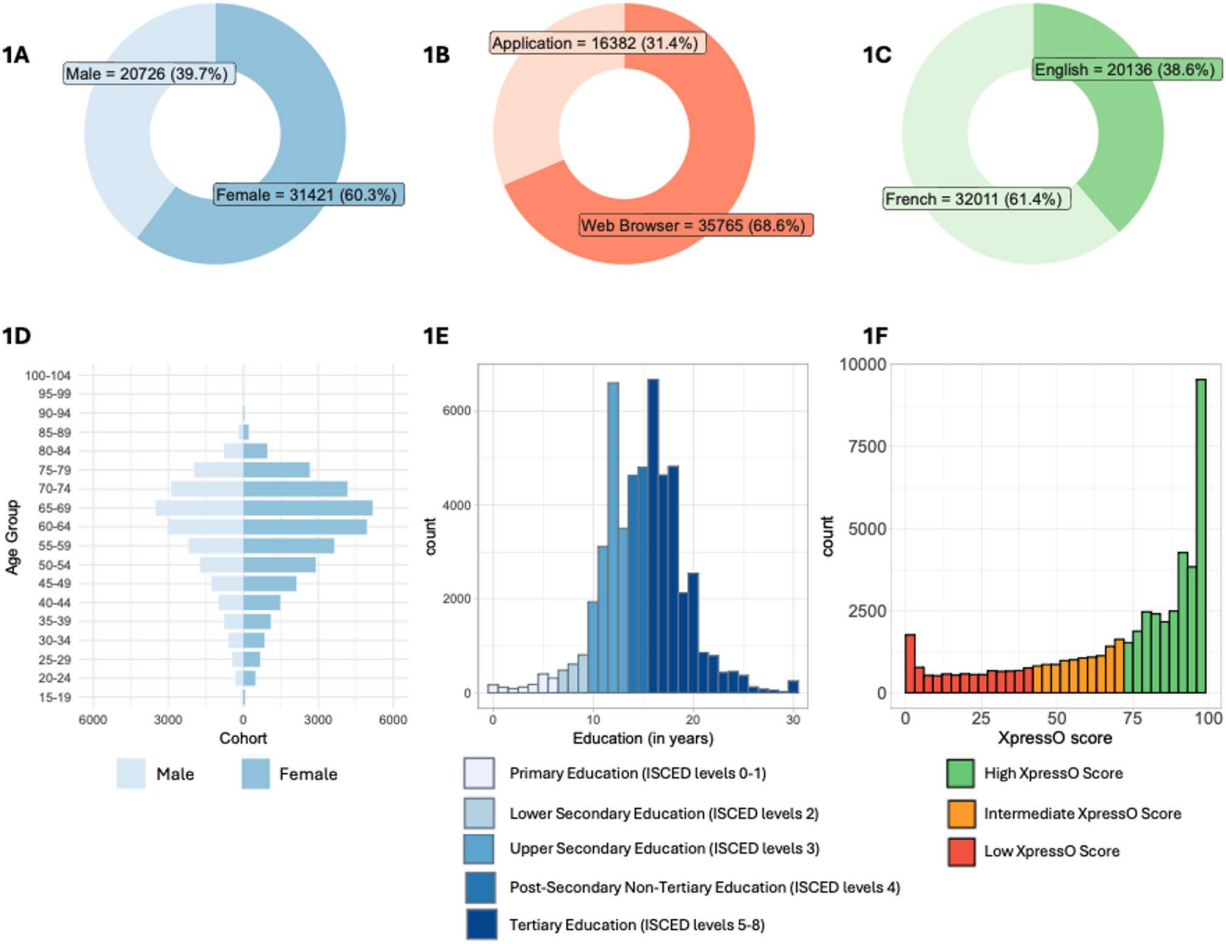
Demographics and scores of online participants. The top panel shows donut plots with (**A**)—sex (male / female), (**B**)—platform (mobile application/web browser), and (**C**)—language of the instructions. The bottom panel shows (**D**)—the distribution of age for each sex, (**E**)—years of education. The years of education are color coded according to the (merged) ISCED levels, and (**F**)—XpressO scores color coded according to the categorical levels.

We analyzed 52,147 participants with an average age of 59.4 years (SD 14.5), 60.3% female vs. 39.7% male, and a mean of 15.0 years of education (SD 4.12) (See Table 1, Fig. 1A). Of these participants, 68.6% accessed the test via a web browser, while 31.4% via the mobile application (Fig. 1B), and 61.4% completed the XpressO test in French and 38.6% in English (Fig. 1C). The age distribution of online participants was centered around 62 years, slightly left-skewed, and similar for men and women (Fig. 1D). Participants under 18 years were excluded and the distributions became progressively sparse with higher age, with very few participants above 90. The distribution of education showed a median of 15 years. The frequencies for 12 and 16 years of education stood out from the histogram (Fig. 1E), consistent with completing high-school or four years of college in the United States. Among the 2,858 of participants who shared their location, 90.9% were in the United States and most used English instructions. Most participants who conducted the test via a web browser were self-recruited following a news article in “Le Journal de Montréal”. This resulted in a recruitement wave that started in early 2025 (see Supplementary Fig. 3 and 4). To account for the recruitment wave, we added a nuisance regressor to the statistical models. The online participants in the recruitement wave almost all used French instructions and were most likely located in the French speaking part of Canada. While we do not know the exact location of most participants, the majority appeared to be in North America.

Across the entire test, the average number of correct items on the delayed recall task was 4.81 (out of 5.00), and the average number of 100% correct on the logical task was 6.00 (out of 7.67). The average time to complete the XpressO test was 5.06 min (SD 1.79). The results reported here from the online cohort were based on the updated XpressO model (cf. Supplementary Materials). The distribution of XpressO scores was bimodal, consistent with the S-shaped curve of the logistic regression model (Fig. 1E). In the online cohort, 18.9% of participants exhibited a low XpressO score, 20.9% an intermediate XpressO score, and 60.3% reached a high XpressO score (Table 1). These results suggest that most of the online participants would score in the normal range on a MoCA test.

**Fig. 2:**
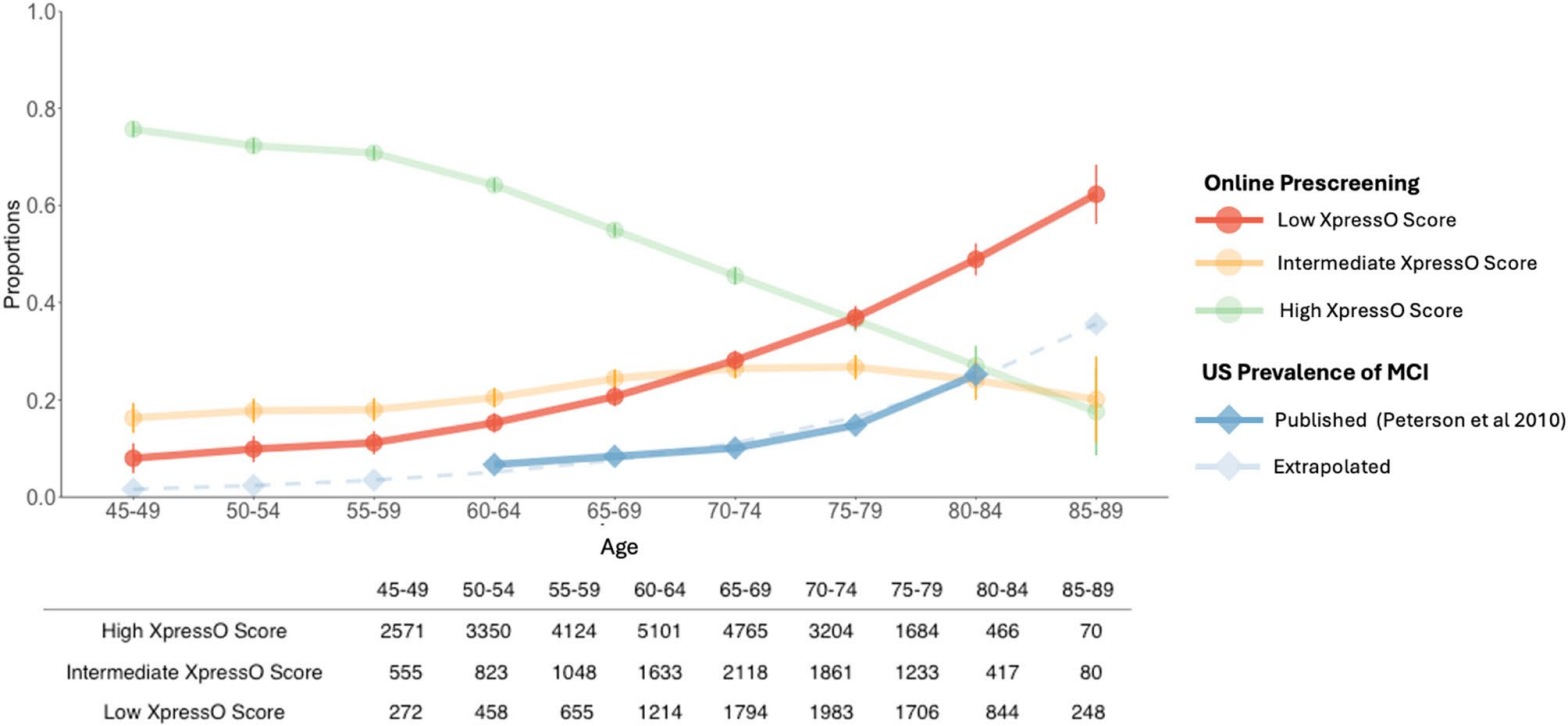
Proportion of low, intermediate and high XpressO scores stratified by age. In green, the proportion of high XpressO scores. In orange, the proportion of intermediate XpressO scores, and in red, the proportion of low XpressO scores, in dark blue, the age-relative prevalence of mild cognitive impairment (MCI) in the United States (US) based on Peterson et al. (2010). The light blue value and dashed line show an extrapolation of prevalence estimates. The x-axis shows age in bins of 5 years. The y-axis the proportion of each categorical group. Error bars reflect the 95 percent confidence interval. The table shows the number of observations (participants) for each age-bin and XpressO score category.

When comparing demographic factors, the median age was 69 for low XpressO scores, 65 for intermediate and 59 for high scores (Table 1). The proportion of female participants was 51.6% and the proportion low XpressO scores was 18.9%, intermediate 58.8%, and high 63.5% The median education was 13 years for low XpressO scores, 15 for intermediate scores and 16 for high scores (Table 1). We visualized the proportion of low, intermediate and high XpressO scores relative to age using 5-year bins (Fig. 2). These resulting curves illustrated that the proportion of low XpressO scores increased with age, especially above 60 years where it roughly doubled every decade. In contrast, the proportion of intermediate XpressO scores increased slightly, but then remained stable.

**Fig. 3:**
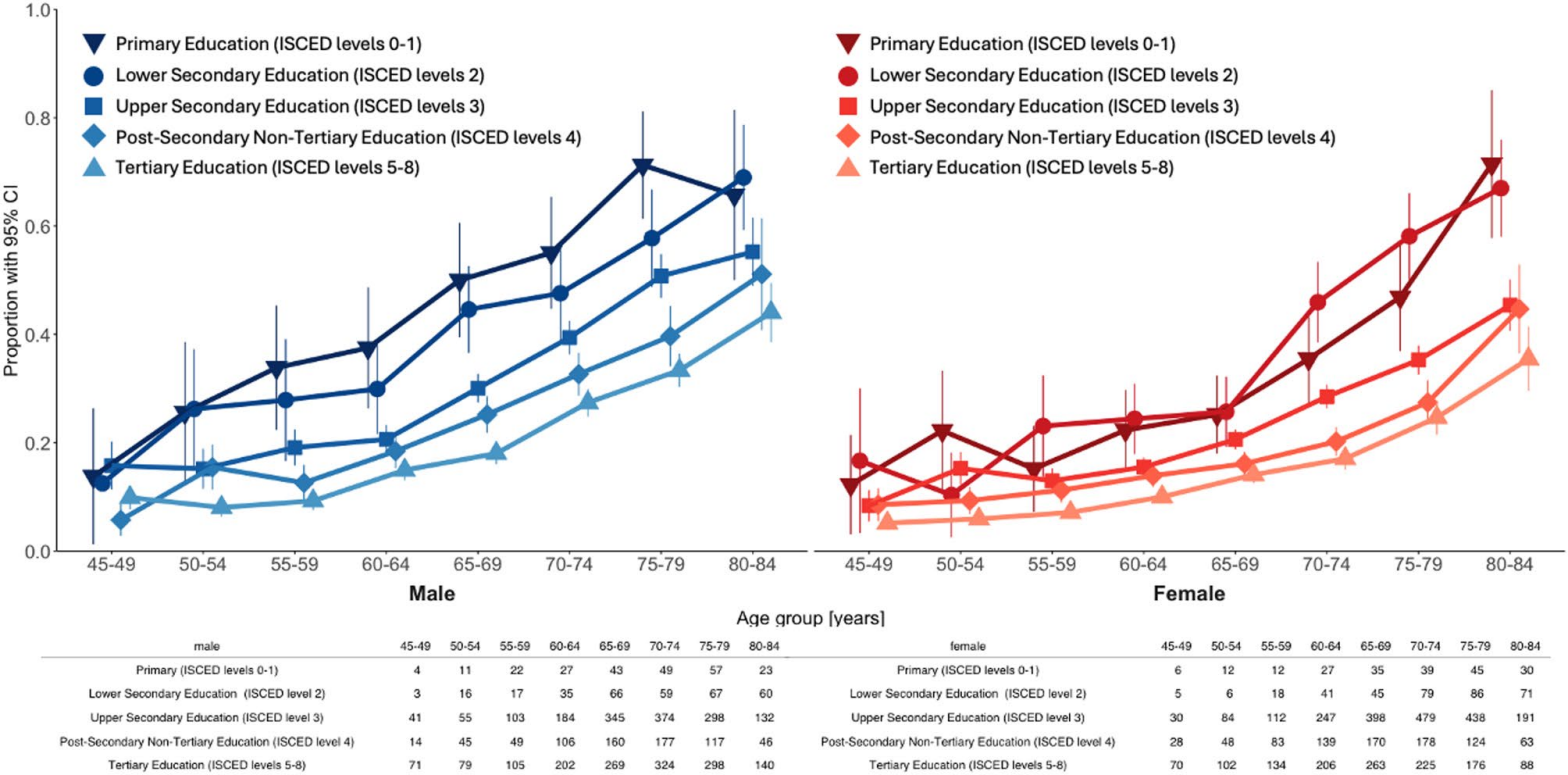
Proportion of Low XpressO scores stratified by sex, age, and education. On the left, in blue, the proportion of low XpressO scores in males. On the right, in red, the proportion of low XpressO scores in females. On the x-axis age in bins of 5 years. On the y-axis the proportion of low XpressO scores. The color intensity and shape reflects the years of education grouped by ISCED levels; the darker face-down triangle indicates ISCED level 0 and 1, the circle level 2, the square level 3, the diamond level 4 and the lighter face-up triangle levels 5–8. Error bars reflect the 95 percent confidence interval. The tables shows the number of observations (participants) with a low XpressO score for each age-bin and each sex.

We also visualized the proportion of low XpressO scores stratified by sex, age, and education, by separately plotting the corresponding fraction of males and females with low scores for five education levels (Fig. 3). These curves showed a clear association between age and increased proportion of low XpressO scores. The curves also showed a y-axis offset that suggested a higher overall positive prescreening rate for males. Strikingly, the curves illustrated the effect of education with an x-axis offset for each additional level of education. The curves also suggested several interactions: A possible interaction between age and education was observed as the age-relative proportions of positive prescreening tests increased more slowly with higher levels of education. The curves also suggested an interaction between sex and education: There was a visible offset in the curves for females that tended to perform better than males at the same education-level. Finally, these curves suggested a three-way interaction: the proportion of low XpressO scores increased more slowly for higher education levels, but this proportion increase appeared to be delayed in females relative to males.

### Effects of sex, age, and education

In the un-adjusted (raw) models, females showed higher XpressO scores than males (*β*_1_ = 5.836; CI_95_ = [5.330, 6.342]; *p*-value < 0.001; AIC = 498,639), we identified a negative association with age (*β*_1_ = − 0.653; CI_95_ = [− 0.670, − 0.636]; *p*-value < 0.001; AIC = 493,619), and we found a positive association with years of education (*β*_1_ = 1.368; CI_95_ = [1.309, 1.428]; *p*-value < 0.001; AIC = 497,139), and a lower XpressO score in the second recruitment wave (*β*_1_ = − 7.125; CI_95_ = [− 7.640, − 6.609]; *p*-value < 0.001; AIC = 498,445). In the models adjusted for sex, age, education, and all interactions with a randon effect term for recruitement wave, females again obtained higher XpressO scores than males (*β*_2_ = 11.61; CI_95_ = [3.788, 19.425]; *p*-value_FDR_ = 0.004; AIC = 491,811), age was again negatively associated with the XpressO score (*β*_1_ = − 0.925; CI_95_ = [− 0.883, − 0.812];* p*-value_FDR_ < 0.001; AIC = 491,811), while education alone showed no significant association with the XpressO score (*β*_3_ = 0.054; CI_95_ = [− 0.311, 0.420]; *p*-value_FDR_ = 0.772; AIC = 491,811), and XpressO score was lower in the second recruitment wave (*β*_4_ = − 0.813; CI_95_ = − 1.322, − 0.304]; *p*-value_FDR_ = 0.002; AIC = 491811]. The interaction effect for age and sex was not significant (*β*_5_ = − 0.061; CI_95_ = [− 0.186, 0.063]; *p*-value_FDR_ = 0.380; AIC = 491,811). In contrast, both interaction effects for age and education (*β*_6_ = 0.017; CI_95_ = [0.011, 0.023];* p*-value_FDR_ < 0.001; AIC = 491,811), and for sex and education (*β*_7_ = − 0.896; CI_95_ = [− 1.386, − 0.405]; *p*-value_FDR_ < 0.001; AIC = 491,811) were significant. The three-way interaction term between age, sex, and education was also significant (*β*_8_ = 0.013; CI_95_ = [0.005, 0.021]; p-value_FDR_ = 0.002; AIC = 491,811). Supplementary Table 4 lists these results with additional metrics including log-likelhood, BIC and residuals. Supplementary Table 3 and 5 provide a comparison with ordinal and robust regression. These comparisons demonstrate that the results are not driven by non-linear residuals or outliers. Finally, when estimating the marginal effects with a model adjusted for all interaction terms and the recruitment wave, we also found that females showed higher XpressO scores than males (*β*_1_ = 5.796; CI_95_ = [5.321, 6.271]; *p*-value < 0.001), we again identified a negative association with age (*β*_1_ = − 0.591; CI_95_ = [− 0.608, − 0.574]; *p*-value < 0.001), and we found a positive association with years of education (*β*_1_ = 0.998; CI_95_ = [0.939, 1.056]; *p*-value < 0.001).

### Potential confounders

In un-adjusted regressions, we found that the XpressO score was lower in participants using the web browser (*β*_1_ = − 6.293; CI_95_ = [− 6.827, − 5.760]; p-value < 0.001; AIC = 498,615) and that it was higher in participants using English instructions (*β*_1_ = 6.997; CI_95_ = [6.489, 7.505]; *p*-value < 0.001; AIC = 498,422). In regressions adjusted for demographics, there was no significant difference in the XpressO score between using a web browser or the mobile application (*β*_1_ = − 0.124; CI_95_ = [− 0.731, 0.483]; *p*-value_FDR_ = 0.746), and language showed a much smaller and reversed effect, with lower XpressO scores in participants using English instructions (*β*_2_ = − 1.297; CI_95_ = [− 1.896, − 0.697]; *p*-value_FDR_ < 0.001). These results indicate that the observed differences for individuals using the mobile application or using the French version were (mainly) due to demographic differences.

### Effect of demographic adjustments on prescreening performance

In the clinical validation cohort, see Supplementary Materials clinical study 2, the AUC for the unadjusted XpressO score was 0.860 for predicting low MoCA scores. The AUC of the score adjusted for sex, age, and education was slightly lower (AUC = 0.814). A DeLong test comparing the two AUCs confirmed that the AUC difference was statistically different (Z = 2.418, CI_95_ = [0.009, 0.084], *p*-value = 0.016). Also, the AUC of the logistic score adjusted for sex, age, education, and the interaction terms was slightly lower (AUC = 0.816). Again, a DeLong test confirmed that the AUC was statistically different (Z = 2.313, CI_95_ = [0.007, 0.082], *p*-value = 0.021). These results confirmed that the demographic adjustment is detrimental for the discrimination power of prescreening tests for cognitive impairment.

## Discussion

In this study, we presented real-world evidence for online prescreening of cognitive impairment. We quantified the associations between, sex, age, and education, including the interactions. We found that adjusting for demographic factors reduces the observed effect of the potential confounders—platform and language—although this adjustment did not improve the prescreening performance. Below, we provide context, interpret these results in the context of prevalence of cognitive impairment, and discuss the broader implications for online cognitive prescreening.

The real-world online cohort of 50,000 adults comprised more women, was more highly educated and older than the general population of North America: The population of the United States is 51.5% female / 48.5% male, with an average age of 39.2 years, and a median of 13 years of education^47^; In Canada, the population is 51.3% female / 48.7% male, the average age is 41.9 years, with the same median of 13 years of education^48^. This over-representation of older persons, women, and individuals with higher levels of education is typical for studies on subjective cognitive decline^49,50^, and it has also been seen in clinical trials on early treatment for Alzheimer’s disease^51^. Therefore, we can assume that the online cohort is enriched with older adults who are concerned about their brain health.

The observed increase of the proportion of adults with a low XpressO scores with age is also consistent with the age-related increase in the prevalence of (mild) cognitive impairment or dementia reported in the United States^52^. When comparing the proportions in the age ranges from 65–69 and 80–84, the US prevalence of MCI increases threefold from 0.084 to 0.252, while the observed proportion of Low XpressO scores increased twofold from 0.207 to 0.489 (see Fig. 2). Overall, the XpressO score predicts the same trend, but about twice as many cases of cognitive impairment in this online cohort, as would be expected in the general population. This could indicate that the cohort contains more individuals with (subjective) cognitive impairment, consistent with an increased interest in brain health. Alternatively, the reported MCI prevalence may have been underestimated, or other factors may explain the increased proportion of low XpressO scores. For example, in the 35–40 age-range, the proportion of low XpressO scores was 0.088 (Fig. 2), while the true prevalence of cognitive impairment in this age-range is tiny. Thus, some online participants might not have completed the XpressO task as seriously as in a supervised clinical care or population screening setting.

The directions of the observed associations between sex, age, and education with the XpressO score are consistent with the previously established associations between demographic factors and the paper MoCA total score^26,27,32^. Noting that the XpressO score reflects a logistic probability, scaled from 0–100 for prescreening positive for (mild) cognitive impairment or dementia on the MoCA^21^, we can thus interpret the model estimates as the risk of screening “positive”. The marginal estimates indicated that one year of aging coincides with a 0.65% increase in the relative risk for prescreening positive, women had lower relative risk by 5.8% and each additional year of education reduced the relative risk by 1.37% . The directions of the raw and marginal estimates were consistent with the main effects in the linear model that included the interaction terms between sex, age, and education. Thus, although main effects can be hard to interpret in the presence of interactions, age and education were confirmed as risk and protective factors, respectively, and an additional level of education, equivalent to four years of training, “compensates” for half a decade of aging.

**Fig. 4:**
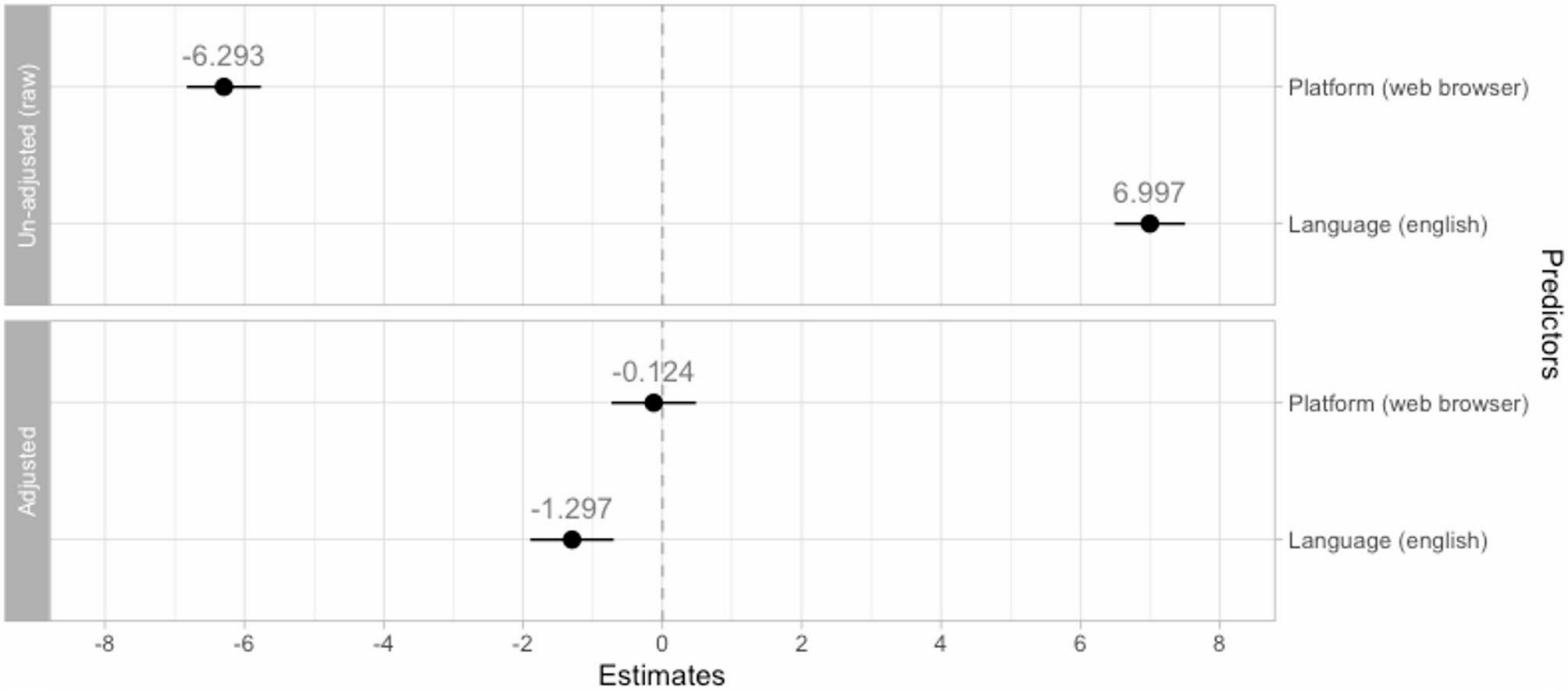
Estimates of potential confounders before and after adjusting for demographics. On top, the un-adjusted (raw) estimates before correction, on the bottom the estimates after a demographic adjustment. On the x-axis, the coefficient weight, that can be interpreted in units of the XpressO score. On the y-axis the potential confounders (platform and language). The black dot indicates the point estimate, and the bars reflect the 95% confidence interval. In grey, the numerical value annotates the point estimate.

Up to the end of 2024, most participants completed the online XpressO test with English instructions, even though French had also been made available at the same time. After XpressO was promoted in a news article in “Le Journal de Montréal” [https://www.journaldemontreal.com/2025/01/06/etes-vous-prets-a-tester-votre-memoire], many new participants performed the test using French instructions, and almost all of them completed it in a web browser. After we pooled the data, we found that participants who had completed the XpressO test in a web browser scored 6 points lower than in the app (see Fig. 4). Also, we found that users of the English instructions scored 7 points higher. To clarify, we explored the influence after adjusting for demographics: We found that the demographics accounted for (and thus removed) the influence of the platform (Fig. 4), and greatly reduced the effect size for language. This residual language-related effect was now inverted, indicating that participants with English instructions scored 0.91 points lower. We have no clear explanation for this, but speculate that some participants that used the English instructions might not have been able to select their true native language. This could result in slightly lower scores. In the future, when more data from additional languages becomes available, we will further investigate the influence of the instructions and see if this effect remains.

In line with a recent simulation study^20^, we found that a demographic correction slightly reduced the discrimination performance of the XpressO score, as the AUC was reduced by approximately 0.045 in our clinical cohort. This does not mean that a correction of the XpressO score is not useful. As illustrated by the correction for potential confounders, a correction can reassign or reattribute the influence of a confounder, while a causal model would be required to disambiguate.

This study has several limitations. First, the clinical cohort that we enrolled was of moderate size, and did not include many participants at the more extreme strata (e.g. very low education, or very old males, etc.). In fact, the clinical cohort was enrolled at the same clinic that was used in the development of the XpressO test. Additionally, the clinical cohort lacked ethnic diversity, as most participants were white and relatively highly educated. This homogeneity may limit the generalizability of some of the findings to more diverse populations or other geographical locations. Second, although we found that demographic factors can account for most of the differences in language and platform, these factors can also coincide with other un-measured factors and provide a proxy for geographical regions or language proficiency. Thus, the residuals effects of platform and language might be over-or underestimated and not simply generalize to other populations. Third, the real-world online cohort is self-enrolled and likely enriched with participants who are concerned about cognitive decline and brain health. Also, in the online cohort. we excluded participants who did not disclose age, education, sex, or reported sex as “other”. Both factors may have introduced sampling biases and warrant caution when generalizing results to other settings, especially regarding differences between males and females. In future updates of XpressO, we plan to gather additional information on gender, ethnicity, and geographical location, so we can better characterize the cohort and evaluate how these characteristics influence prescreening results. A final limitation follows from the XpressO score that is designed detect the differences between cognitively normal adults and MCI. It is therefore not optimized to detect more subtle cognitive decline in the range of cognitively normal older adults. In future studies, we will investigate longitudinal prescreening results in order to provide more precise estimates and enable earlier detection of cognitive decline.

In conclusion, these results provide novel understanding of the influence of demographic factors on prescreening for cognitive impairment. The results demonstrate that sex, age, and education interact in a complex manner, effecting both the prevalence of the disease and the XpressO test score. Specifically, men are more likely to prescreen positive for (mild) cognitive impairment or dementia. In contrast, education has a strong protective effect. In general, the results demonstrate that XpressO can be used for online prescreening, and thereby, help identify individuals that require a more in-depth clinical evaluation. In that manner, prescreening can help provide faster and more equitable access to healthcare for patients, and identify patients early on who might be eligible for disease-modifying treatment for Alzheimer’s disease.

## Supplementary Information

Below is the link to the electronic supplementary material.

Supplementary Material 1

## Data Availability

Data will be available upon request via after a data transfer/sharing agreement. For contact details see https://mocacognition.com/contact.
