# Supplementary material for: Real-world evidence from 50,000 online participants using MoCA-XpressO for cognitive prescreening
Source: Sci Rep. 2026 Jan 13;16:5092. doi: 10.1038/s41598-026-35640-0 (PMC12877080; doi:10.1038/s41598-026-35640-0)
Supplement: Supplementary file 1 — Supplementary Material 1 [file 41598_2026_35640_MOESM1_ESM.pdf]

Supplementary Material

After its release in early 2024, the XpressO application has undergone several minor updates to accommodate Android and iOS system updates, improve usability [1], support additional languages, and execution in a web browser. In parallel, we updated and re-validated the logistic regression model using data from two clinical studies. In clinical study 1, we included 108 patients and caregivers at the MoCA Clinic who were first assessed by a rater-administered MoCA test followed by a digital XpressO test in an unsupervised setting (see Supplementary Table 1). The test order was fixed based on findings from the original study indicating no significant influence on XpressO results from conducting a prior MoCA test [2]. The second study included 101 patients and caregivers from the MoCA Clinic, as well as older adults from a retirement home near Montreal (see Supplementary Table 1), and used an identical design. The MoCA tests were administered in French and included several versions (1.0% v7.1; 1.0% v7.2, 79.2% v8.1; 8.9% v8.2 and 8.9% v8.3). The participants, and technicians who administered and scored the MoCA , did not see the results of the XpressO test.

We used the cohort from the first clinical study, to update the XpressO logistical regression score (see Supplementary Table 1). In this first cohort, we repeated analyses from the original study [2]: we applied a lasso regression to evaluate the features used to predict the MoCA cut-off for cognitive impairment (MoCA total score ≤24/30). The lasso regression removed 2 out of 5 of the original features: the average number of drags on the memory task and the average number of drags in the logical task. This was consistent with the small weighting for these drags in the original regression model. Hence, we updated the weights of the logistic regression model using the three remaining features: the average number of same objects on the memory task, the average number of 100%-correct answers on the logical task and the total time (see Supplementary Table 1). Subsequently, we validated the updated logistic regression model using the cohort from the second clinical study (see Supplementary Table 1).

In both studies, we compared the original logistic score with the updated XpressO score using a Spearman correlation, visualized the relation with a scatter plot and fitted a linear model (see Supplementary Figure 1). Secondly, we calculated the area under the curve (AUC) of the XpressO score relative to the MoCA cut-off for cognitive impairment, MoCA total score ≤24/30 [3]. We also compared the AUC of the original logistic score and the updated score using a DeLong test for two correlated ROC curves. Next, we visualized the relation between the updated XpressO score from the updated model and MoCA total score in a scatter plot and fitted a 3rd degree polynomial. We also categorized participants into low, intermediate, or high XpressO scores based on the cut-offs of 42 and 72 respectively [2]. Additionally, we evaluated the validity of the cut-offs by fitting a linear model stratified by low, intermediate, and high XpressO score and calculated stratified correlation coefficients. In the main analysis for the online cohort, we only showed results from the updated model.

Follow-up analyses

We conducted several follow-up analyses to provide additional insights. First, we estimated the sensitivity/specificity curves and calibration curves for both clinical studies (Supplementary Figure 2). The calibration curves used 50-fold bootstrapping and estimated the frequencies across six equally spaced bins. For each bin, we estimated the mean and the 95% confidence interval (CI) using the 2.5 and 97.5 percentiles.

Second, in the clinical validation study, we evaluated the influence of the education correction on the MoCA total score. The MoCA was administered according to current guidelines and included a one-point adjustment for participants with 12 years of education or less. In 3 out of 101 participants, this influenced the ground-truth label, as the MoCA total scores dropped from 24 to 23 without the adjustment. One participant each was equally distributed in the low, intermediate and high XpressO categories. To evaluate the influence of the education adjustment, we estimate the AUC, Brier score, sensitivity and specificity for both XpressO score cut-offs, 42 and 72, with and without the education correction (Supplementary Table 2). The Brier score was defined as the mean squared difference between predicted probabilities and the binary outcomes (MoCA total

score ≤24/30). The XpressO scores was rescaled to a range of 0-1 representing the predicted probability of a MoCA total score ≤24/30. The Brier score was then calculated as the mean of the squared differences between the predicted probabilities and the observed (binary) outcomes.

Third, we evaluated the effects of age using non-linear models. We visualized the age-related curves using Generalized Additive Models (GAMs) separately for males and females. Additionally, we estimated incorporating age as a smooth function and stratified by sex and education using categorical ISCED levels. Only main effects are reported, as non-linear interactions are difficult to interpret.

Fourth, we evaluated an ordinal least squares regression. The XpressO Score is a logistic probability bounded between 0 and 100, and as a dependent variable in a linear regression, it may violate the assumptions regarding homoscedasticity and normally distributed residuals. Therefore, we estimated ordinal regression models using low, intermediate and high XpressO categories and compared the results with the standard GLM regression. Using both methods, we estimated the raw effects and the full model with age, sex, and education along with all interaction terms. Recruitment wave was included as a covariate to adjust for potential confounding effects (Supplementary Tables 3 and 4).

Fifth, to further clarify the influence of non-linear residuals, and/or outliers, we also evaluated a robust regression of the full model with age, sex, and education, along with all interaction terms and recruitment-wave as a covariate. Again, we fitted a generalized linear model predicting XpressO scores using age, sex, education, and their two- and three-way interaction terms. To account for potential heteroskedasticity and improve the reliability of inference, we computed robust standard errors using the HC1 estimator from the sandwich package in R. This approach allows for more accurate estimation of coefficient uncertainty in the presence of non-constant variance or influential observations. Supplementary Table 5, shows these robust estimates together with the standard least squares regression.

Sixth, we evaluated the influence of recruitment period. Following a news-article in the French Canadian “Le Journal de Montréal”, many new participants self-enrolled via the website. We plotted the number of XpressO sessions per week together with the proportion high, intermediate and low XpressO scores, to show the recruitment wave (Supplementary Figure 3). In addition, we visualized the proportion of high, intermediate and low XpressO score by age, before and after the recruitment wave (Supplementary Figure 4). To mitigate the influence of the recruitment wave, we have added a factor term in the models, as a covariate to account for a bias.

Seventh, to provide further insight into the real-world cohort, we also estimated the positive predictive value (PPV) and false omission rate (FOR) stratified by age (7Supplementary Table 6). The proportion of participants with a Low XpressO Score within each age group was used as a proxy for the prevalence of a positive prescreening. PPV was defined as the estimated probability that individuals with a Low XpressO Score would truly have mild cognitive impairment (MCI), assuming the sensitivity/specificity observed in the clinical validation study. FOR was defined as the estimated probability that individuals without a Low XpressO Score might still have MCI, and was calculated as one minus the negative predictive value (NPV). This approach allowed us to approximate prescreening performance in the online real-world cohort in the absence of ground-truth diagnosis and provided relevant PPV and FOR estimates for pre-screening with XpressO. However, given that these online estimates were higher than the prevalence of MCI in the United States (US), we also estimated the PPV and FOR based on published US prevalence numbers of MCI between ages 60 and 85 (Peterson et al 2010).

Supplementary Results

Supplementary Table 1 shows the demographics, task metrics, logistic regression scores ("XpressO score") and the MoCA total scores for both clinical studies. In the first study, 63.0% had a MoCA total score ≤24/30 consistent with (mild) cognitive impairment or dementia, and in the second study the value was slightly lower, at 60.4%. A chi-squared test indicated that these proportions are not significantly different ($\chi^2 = 0.0572$, p-value = 0.811). Supplementary Figure 1 shows the results of the updated logistic regression model compared to the original model and the MoCA total score. In the first study, the Spearman rank correlation (*ρ)* between original XpressO score and the updated XpressO score was $\rho = 0.994$ ($p < 0.001$), the correlation between the MoCA total score and the updated XpressO score was $\rho = 0.645$ ($p < 0.001$), and the AUC was 0.885 for classifying cognitive impairment. In the second study, the correlation between original XpressO score and the updated XpressO score was $\rho = 0.990$ ($p < 0.001$), the correlation between the MoCA total score and the updated XpressO score was $\rho = 0.695$ (p-value < 0.001) and the AUC was 0.860. These areas under the curve are slightly higher than the AUC = 0.845 from the original, somewhat smaller study [2]. We also compared these AUCs from the original and updated model using a DeLong test. For the first study, we found Z = 0.230, CI_{95} = [-0.011, 0.014], p-value = 0.82, and for the second study, we found Z = 1.080, CI_{95} = [-0.023, 0.007], p-value = 0.28. Thus, in both studies we did not find a significant difference in performance compared to the original model.

In the first study, we found stratified Spearman rank correlations of $\rho_{low} = 0.270$ (p-value = 0.040), $\rho_{intermediate} = -0.066$ (p-value = 0.790), and $\rho_{high} = 0.308$ (p-value = 0.076), and in the second study, we found $\rho_{low} = 0.529$ (p-value < 0.001), $\rho_{intermediate} = 0.049$ (p-value < 0.850) and $\rho_{high} = 0.372$ (p-value < 0.061). These stratified coefficients, along with the 3rd degree polynomial, support retaining the previously established cut-offs for low, intermediate, and high scores [2].

Supplementary Table 1: demographics and scores from clinical studies

	Clinical Study 1 (N=108)	Clinical Study 2 (N=101)
**Age (years)**		
Mean (SD)	67.9 (9.68)	73.0 (11.0)
Median [Min, Max]	69.0 [26.0, 86.0]	76.0 [37.0, 93.0]
**Sex**		
Male	46 (42.6%)	41 (40.6%)
Female	62 (57.4%)	60 (59.4%)
**Education (years)**		
Mean (SD)	13.5 (3.62)	13.9 (4.31)
Median [Min, Max]	14.0 [0, 22.0]	14.0 [1.00, 37.0]
**Memory tasks: Average number of same objects**		
Mean (SD)	4.44 (0.698)	4.50 (0.671)
Median [Min, Max]	4.67 [2.00, 5.00]	4.67 [1.67, 5.00]
**Logical tasks: Average number of 100% correct answers**		
Mean (SD)	4.58 (2.18)	4.28 (2.19)
Median [Min, Max]	4.17 [0.333, 7.67]	4.00 [0.333, 7.67]
**Total time (minutes)**		
Mean (SD)	6.25 (2.13)	6.55 (2.44)
Median [Min, Max]	5.63 [3.45, 13.0]	5.92 [3.08, 14.7]
**XpressO Score (original)**		
Mean (SD)	41.5 (35.3)	40.6 (32.5)
Median [Min, Max]	34.6 [0.0, 92.8]	31.9 [0.0, 93.3]
**XpressO Score (updated)**		
Mean (SD)	42.2 (37.1)	39.8 (34.3)
Median [Min, Max]	34.1 [0.0, 98.2]	32.6 [0.0, 98.6]
**MoCA Total Score**		
Mean (SD)	22.7 (3.95)	23.1 (3.92)
Median [Min, Max]	23.0 [11.0, 30.0]	24.0 [13.0, 30.0]

Supplementary Figure 1. Validation of updated model

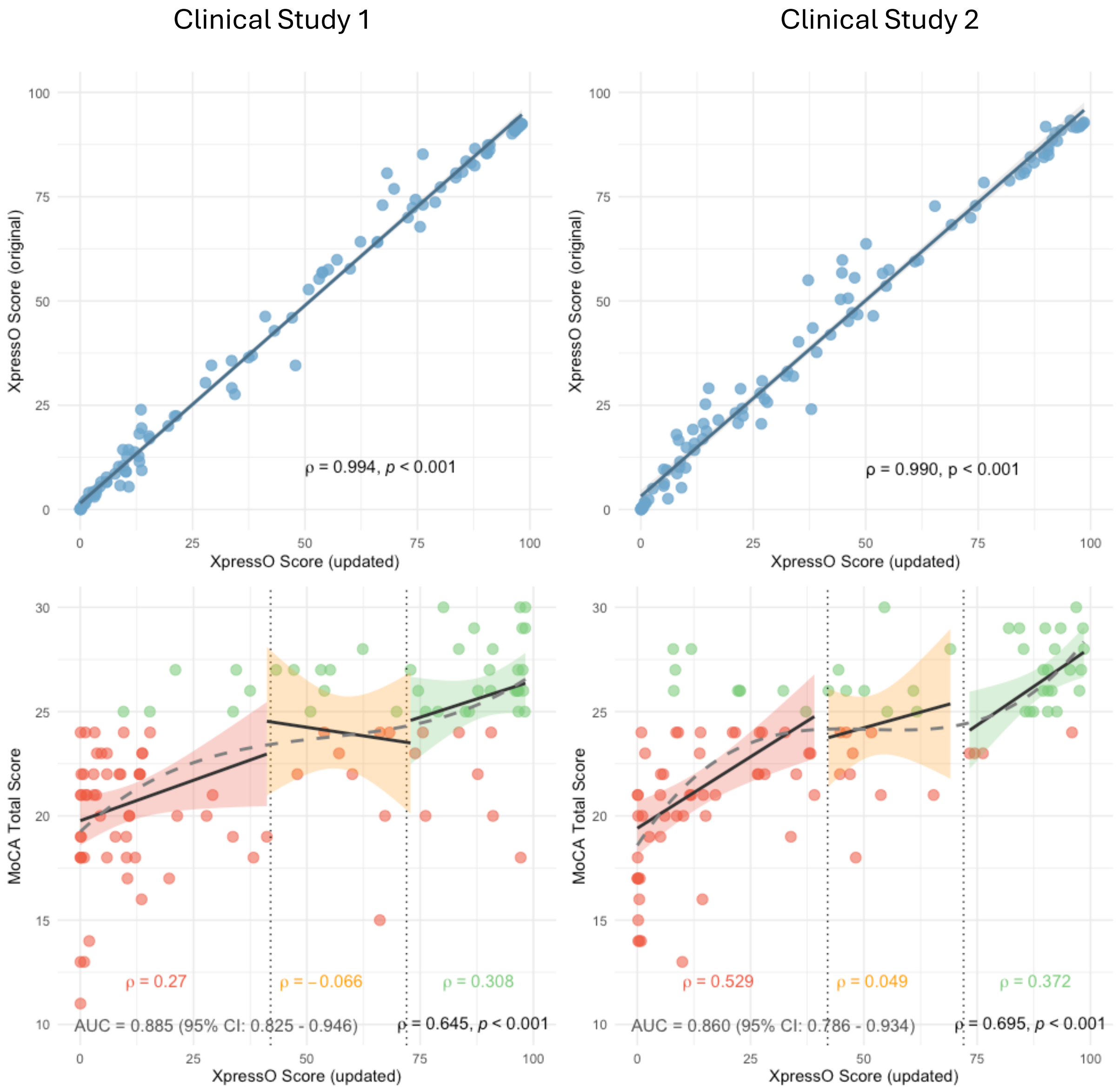

Supplementary Figure 1: The upper panels show scatterplots between the original and updated XpressO scores, including correlation coefficients (ρ) and p-values. The lower panels show scatterplots between the MoCA total score and the updated XpressO score. These include a line fitted separably for low (red), intermediate (orange), and high XpressO scores (green), as well as corresponding correlation coefficients. The dots are color-coded according to the MoCA total score cut-off for cognitively normal (green) versus mild cognitive impairment or dementia (red). The left column shows data from the clinical study 1), used for updating the model weights. The right column shows data from clinical study 2, used for independent validation.

Results follow-up analyses

Supplementary Figure 2. Sensitivity, Specificity, and Calibration curves

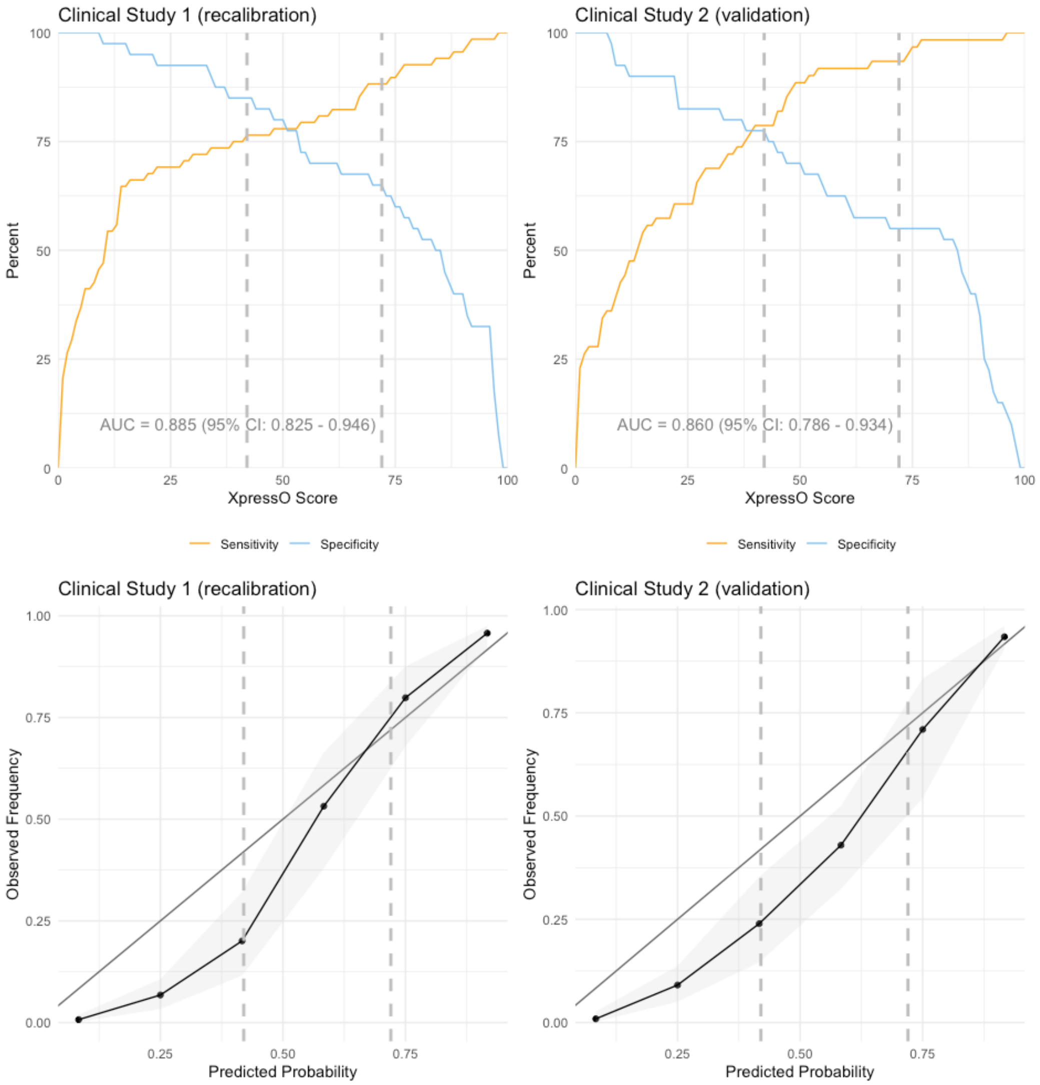

Supplementary Figure 2: The upper panels show sensitivity/specificity curves for both clinical studies. The x-axis shows the XpressO score and the y-axis the sensitivity/specificity from 0 to 100%. The orange line shows sensitivity, the blue line specificity, and the vertical gray lines indicate the cut-off of 42 and 72, Area under the Curve (AUC) and 95% Confidence Interval (CI) are also shown. The lower panels show calibration curves for both clinical studies. On the x-axis, the predicted probability as a percentage. On the y-axis the observed frequency, diagonal indicates the identify line, the dashed vertical gray lines indicate calibration at the cut-off of 42 and 72.

Supplementary Table 2. Influence of education-correction

MoCA	XpressO cutoff	sensitivity	specificity	brier	AUC	CI_{95} low	$CI_{95\ high}$	MoCA ≤ 24	MoCA > 24
Standard MoCA	42	0.787	0.775	0.144	0.860	0.786	0.934	61	40
Standard MoCA	72	0.934	0.550	0.144	0.860	0.786	0.934	61	40
No education correction	42	0.766	0.784	0.151	0.845	0.765	0.925	64	37
No education correction	72	0.922	0.568	0.151	0.845	0.765	0.925	64	37

Supplementary Table 3: non-linear model of age

Covariate	Beta Estimate	[95%-CI]	p-value
Lower Secondary Education (ISCED level 2)	0.16	[-1.67, 2,00]	0.864
Upper Secondary Education (ISCED level 3)	9.11	[7,65, 10.56]	< 0.001
Post-Secondary Education (ISCED level 4)	13.47	[11.98, 14.96]	< 0.001
Tertiary Education (ISCED levels 5-8)	16.83	[15.39, 18.28]	< 0.001
Sex (female)	5.24	[4.78, 5.71]	< 0.001
Recruitement Wave (year)	-3.16	[-3.89, -2.42]	< 0.001
Language (English(	-1.82	[-2.54, -1.11]	< 0.001
Platform (web browser)	1.15	[0.51, 1.79]	< 0.001

GAM: xpresso_score ~ edu_group + sex + s(age, by = sex) + recruivement_wave + language + platform

AIC = 489850

Supplementary Figure 3: non-linear curves of age by sex

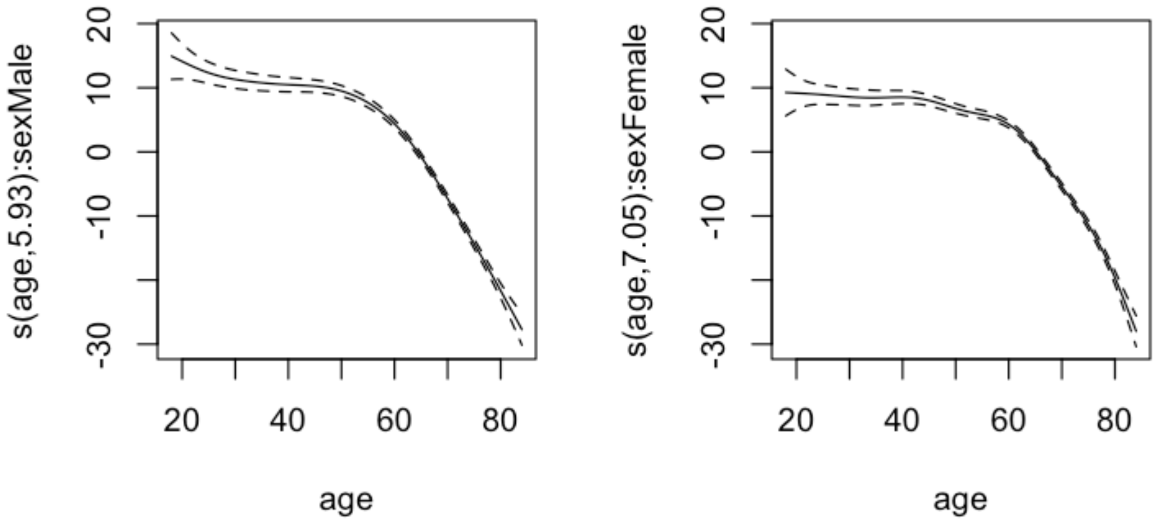

Supplementary Figure 3: The x-axis shows age and the y-axis the XpressO score. O n the left for males and on the right for females. The dotted line indicate the 95% Confidence Interval (CI).

Supplementary Table 4. Ordinal Regression

term	Estimate [CI]	std.error	statistic	p-value	p-value$_{FDR}$	AIC	BIC	logLik	df.residual	model
Age (years)	-0.049 [-0.050, -0.047]	0.001	-65,41	< 0.001	< 0.001	93760	93787	-46877	52144	Raw Estimates
Sex (female)	0.360 [0.326, 0.395]	0.018	20.44	< 0.001	< 0.001	98334	98361	-49164	52144	Raw Estimates
Education (years)	0.090 [0.085, 0.094]	0.002	40.43	< 0.001	< 0.001	97042	97068	-48518	52144	Raw Estimates
Language (english)	0.444 [0.408, 0.480]	0.018	24.43	< 0.001	< 0.001	98144	98170	-49069	52144	Raw Estimates
Platform (web browser)	-0.387 [-0.424, -0.349]	0.019	-20.22	< 0.001	< 0.001	98334	98360	-49164	52144	Raw Estimates
Recruitement Year (2024/2025)	-0.450 [-0.486, -0.413]	0.019	-24.27	< 0.001	< 0.001	98149	98176	-49072	52144	Raw Estimates
Age (years)	-0.047 [-0.050, -0.045]	0.001	-42.29	< 0.001	< 0.001	92382	92470	-46181	52137	Full Model
Sex (female)	0.376 [0.338, 0.413]	0.019	19.76	< 0.001	< 0.001					Full Model
Education (years)	0.071 [0.064, 0.078]	0.003	20.32	< 0.001	< 0.001					Full Model
Recruitement Year (2024/2025)	-0.062 [-0.102, -0.023]	0.020	-3.11	0.001	0.002					Full Model
Age * Sex	0.005 [0.002, 0.008]	0.001	3.46	< 0.001	0.001					Full Model
Age * Education	0.000 [0.000, 0.001]	0.000	1.05	0.292	0.292					Full Model
Sex * Education	-0.008 [-0.017, 0.001]	0.005	-1.76	0.078	0.089					Full Model
Age * Sex * Education	0.001 [0.000, 0.002]	0.000	2.40	0.016	0.022					Full Model

Supplementary Table 4: Results from the ordinal regression model. The upper section shows the raw estimates for each predictor separately. The lower section shows the estimates from the full model with all interaction terms. Both the conventional p-value and False Discovery-Rate (FDR) corrected p-value are included.

Supplementary Table 5. GLM Regression

term	Estimate [CI]	std.error	statistic	p-value	p-value$_{FDR}$	logLik	AIC	BIC	df.residual	model
Age (years)	-0.653 [-0.670, -0.636]	0.009	-76.36	< 0.001	< 0.001	-246807	493619	493646	52145	Raw Estimates
Sex (female)	5.836 [5.330, 6.342]	0.258	22.61	< 0.001	< 0.001	-249316	498639	498666	52145	Raw Estimates
Education (years)	1.368 [1.309, 1.428]	0.030	45.25	< 0.001	< 0.001	-248566	497139	497166	52145	Raw Estimates
Language (english)	6.997 [6.489, 7.505]	0.259	27.02	< 0.001	< 0.001	-249208	498422	498449	52145	Raw Estimates
Platform (web browser)	-6.293 [-6,827, -5.760]	0.272	-23.13	< 0.001	< 0.001	-249305	498615	498642	52145	Raw Estimates
Recruitement Year (2024/2025)	-7.125 [-7,640, -6.609]	0.263	-27.09	< 0.001	< 0.001	-249206	498419	498445	52145	Raw Estimates
Age (years)	-0.925 [-1.018, -0.833]	0.047	-19.64	< 0.001	< 0.001	-245851	491722	491811	52138	Full Model
Sex (female)	11.61 [3.788, 19.43]	3,989	2.91	0.003	0.004					Full Model
Education (years)	0.054 [-0.311, 0.420]	0.186	0.29	0.772	0.772					Full Model
Recruitement Year (2024/2025)	-0.813 [-1.322, -0.304]	0.260	-3.13	0.001	0.002					Full Model
Age * Sex	-0.061 [-0.186, 0.063]	0.063	-0.97	0.332	0.380					Full Model
Age * Education	0.017 [0.011, 0.023]	0.003	5.72	< 0.001	< 0.001					Full Model
Sex * Education	-0.896 [-1.386, -0.405]	0.250	-3.58	< 0.001	< 0.001					Full Model
Age * Sex * Education	0.013 [0.005, 0.021]	0.004	3.14	0.001	0.002					Full Model

Supplementary Table 5: Results from the standard GLM model. The upper section shows the raw estimates for each predictor separately. The lower section shows the estimates from the full model with all interaction terms. Both the conventional p-value and False Discovery-Rate (FDR) corrected p-value are included.

Supplementary Table 6. Comparison with Robust Regression

term	Coefficient	Std.Error Standard	Std.Error Robust	t-value Standard	t-value Robust	p-value Standard	p-value Robust
Age (years)	-0.93	0.05	0.06	-19.85	-16.88	< 0.001	< 0.001
Sex (female)	11.77	3.99	4.64	2.95	2.54	0.003	0.011
Education (years)	0.06	0.19	0.21	0.31	0.27	0.76	0.79
Age * Sex	-0.06	0.06	0.08	-1.01	-0.85	0.314	0.395
Age * Education	0.02	0.00	0.00	5.81	5.00	< 0.001	< 0.001
Sex * Education	-0.90	0.25	0.29	-3.59	-3.14	< 0.001	0.002
Age * Sex * Education	0.01	0.00	0.01	3.15	2.71	0.002	0.007

Supplementary Table 6: Comparison between standard and robust regression. The term indicates the predictor from GLM, including all interaction terms. The standard error, t-value, and p-value are shown for both the standard and robust method.

Supplementary Figure 4. Recruitment

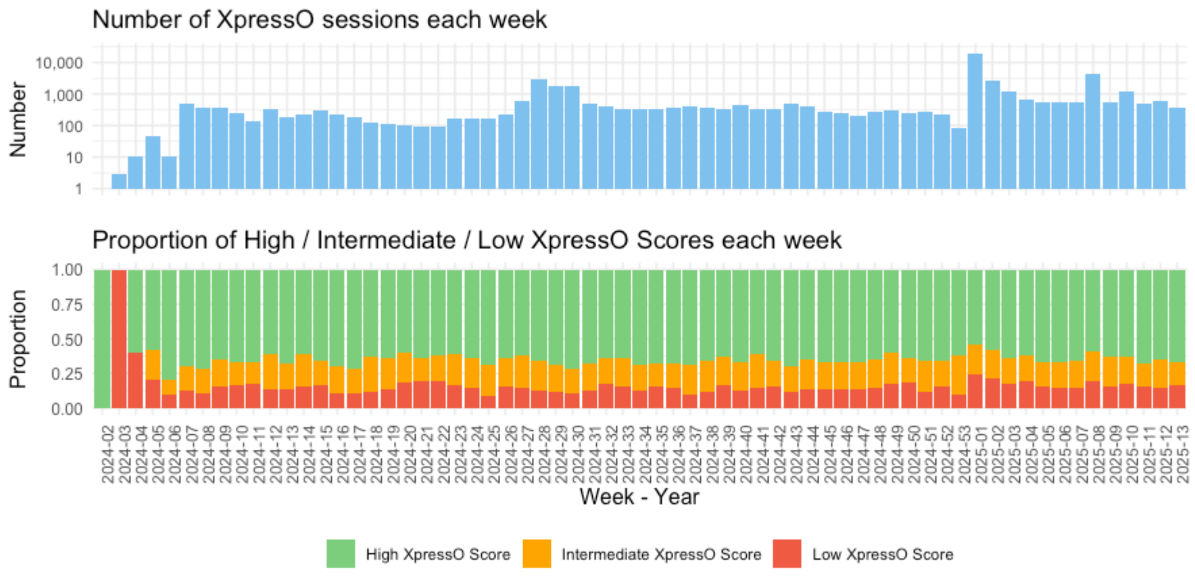

Supplementary Figure 4: The top panel shows the number of XpressO session completed each week by a novel participant. The x-axis indicates the week number, the y-axis the number on a 10 log-scale. Note the peak in recruitment at week 1 of 2025. The bottom panel show the proportion of high, intermediate and low XpressO scores.

Supplementary Figure 5

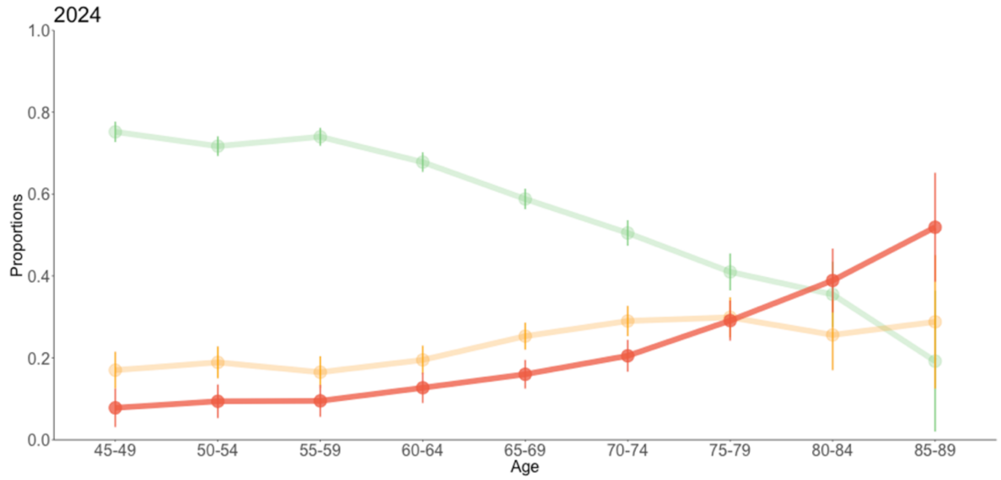

XpressO Score	45-49	50-54	55-59	60-64	65-69	70-74	75-79	80-84	85-89
High XpressO Score	1157	1416	1608	1606	1450	976	467	139	20
Intermediate XpressO Score	262	373	359	462	623	560	340	100	30
Low XpressO Score	120	185	206	302	394	395	332	152	54

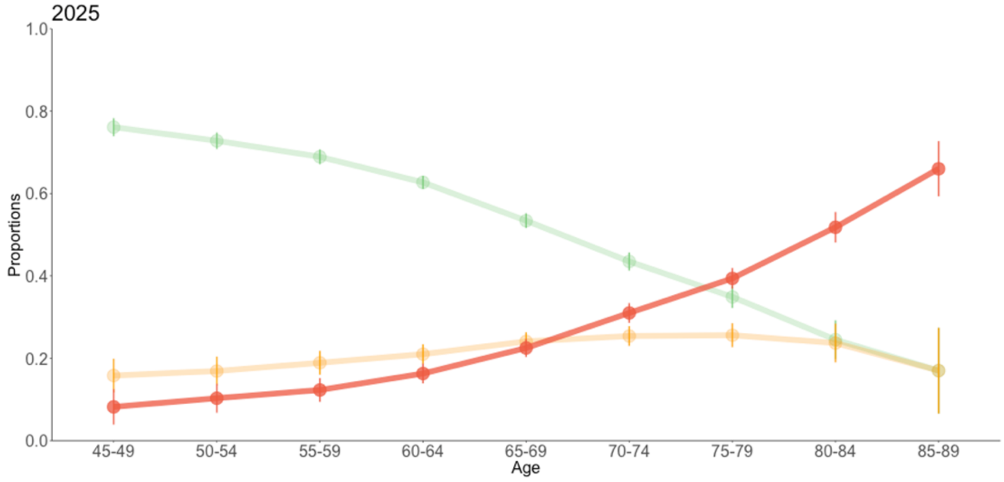

XpressO Score	45-49	50-54	55-59	60-64	65-69	70-74	75-79	80-84	85-89
High XpressO Score	1414	1934	2516	3495	3315	2228	1217	327	50
Intermediate XpressO Score	293	450	689	1171	1495	1301	893	317	50
Low XpressO Score	152	273	449	912	1400	1588	1374	692	194

Supplementary Figure 5: Proportion of low, intermediate and high XpressO scores by age-group before and after the recruitment wave. The top panel shows the curves before the recruitment wave in 2024, and below in 2025. In green, the proportion of high XpressO scores. In orange, the proportion of intermediate XpressO scores, and in red, the proportion of low XpressO scores. The x-axis shows age in bins of 5 years. The y-axis shows the proportion within each categorical group. Error bars reflect the 95 percent confidence interval. The tables show the number of observations (participants) for each age-bin and each XpressO score category.

Supplementary Figure 6

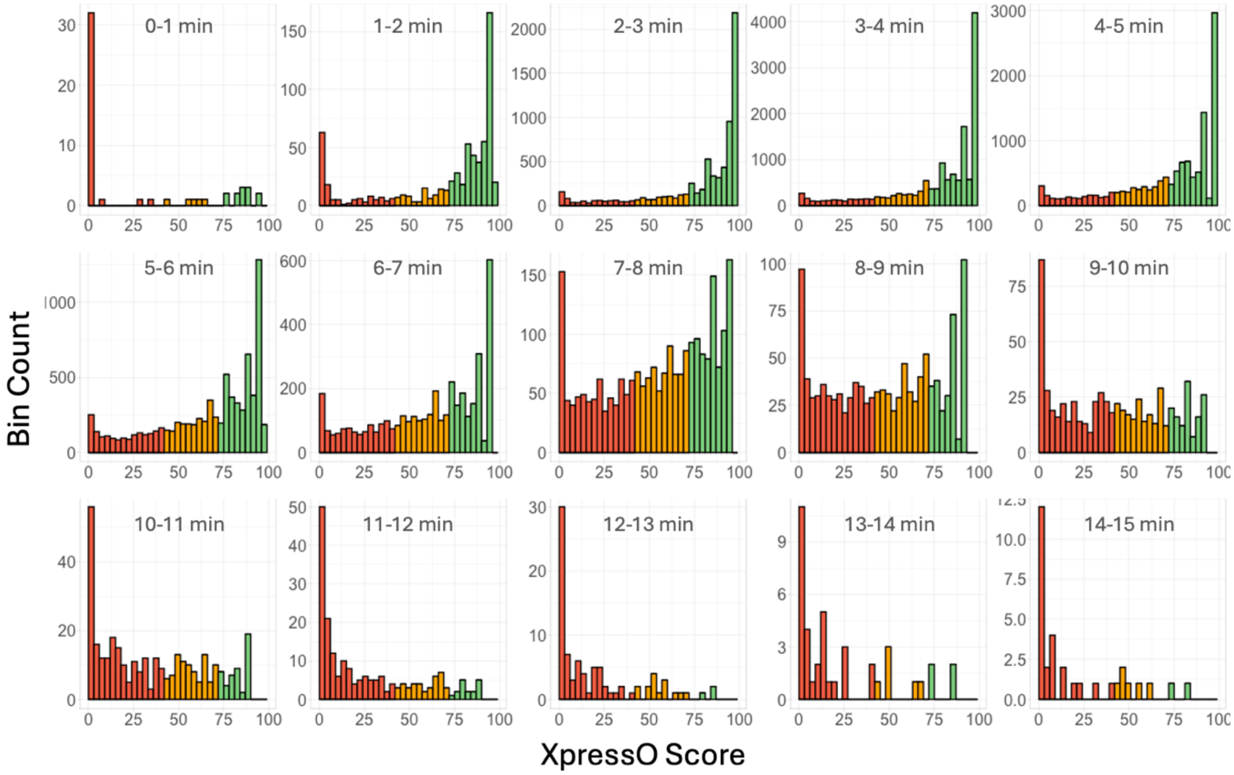

Supplementary Figure 6: Histogram of XpressO scores stratified by total time in minutes (min). In In green, the bin count for high XpressO scores, in orange for intermediate XpressO scores, and in red for low XpressO scores.

Supplementary Table 7

Age	n	proportion low XpressO Score [CI]	PPV (RWE)	FOR (RWE)	PPV (US)	FOR (US)
20-24	785	0.094 [0.027, 0.160]	0.301	0.009		
25-29	1108	0.079 [0.022, 0.136]	0.263	0.007		
30-34	1433	0.072 [0.023, 0.121]	0.244	0.007		
35-39	1854	0.088 [0.045, 0.131]	0.286	0.008		
40-44	2459	0.076 [0.039, 0.113]	0.255	0.007		
45-49	3398	0.080 [0.049, 0.111]	0.265	0.007		
50-54	4631	0.099 [0.072, 0.126]	0.313	0.009		
55-59	5827	0.112 [0.088, 0.136]	0.344	0.011		
60-64	7948	0.153 [0.133, 0.173]	0.429	0.015	0.230	0.006
65-69	8677	0.207 [0.187, 0.227]	0.520	0.022	0.276	0.008
70-74	7048	0.281 [0.261, 0.301]	0.619	0.032	0.318	0.009
75-79	4623	0.369 [0.345, 0.393]	0.708	0.047	0.419	0.015
80-84	1727	0.489 [0.456, 0.522]	0.799	0.075	0.583	0.028
85-89	398	0.623 [0.562, 0.684]	0.873	0.123		

Supplementary Table 7: Age-stratified performance of XpressO. N shows the number of total participants in each 5-year age group. CI provide the 95% confidence interval (CI) of the proportion of low XpressO scores. PPV indicates the estimated positive predictive value in the online real-world evidence (RWE) cohort and the United States (US), based on Petersen et al 2010. FOR indicates the estimated false omission rate for prescreening in the RWE cohort and US, respectively.

Supplementary References

1. Gruber, J. *et al*. Improvements in Usability of MoCA XpressO, a Self-administered Digital Cognitive Pre-screening Tool. *Alzheimer's & Dementia* **20**, e086424 (2024).
2. Klil-Drori, S. *et al*. Montreal Cognitive Assessment (MOCA) XPRESSO : Validation of a digital self-administered cognitive prescreening tool. *J American Geriatrics Society* jgs.18902 (2024) doi:10.1111/jgs.18902.
3. Thomann, A. E., Berres, M., Goettel, N., Steiner, L. A. & Monsch, A. U. Enhanced diagnostic accuracy for neurocognitive disorders: a revised cut-off approach for the Montreal Cognitive Assessment. *Alz Res Therapy* **12**, 39 (2020).
